# Supplementary material for: Ethnic disparities in medication adherence? A systematic review examining the association between ethnicity and antidiabetic medication adherence
Source: PLoS One. 2023 Feb 22;18(2):e0271650. doi: 10.1371/journal.pone.0271650 (PMC9946219; doi:10.1371/journal.pone.0271650)
Supplement: S3 Table — (DOCX) [file pone.0271650.s003.docx]

S3 Table. Results of the risk of bias assessment of retrospective database studies

| Author, year | I | | | | II | | III | | | | VI | | | | | | | | | | | | | | | | | | | | | | | | | V | | | | | | | |
| --- | --- | --- | --- | --- | --- | --- | --- | --- | --- | --- | --- | --- | --- | --- | --- | --- | --- | --- | --- | --- | --- | --- | --- | --- | --- | --- | --- | --- | --- | --- | --- | --- | --- | --- | --- | --- | --- | --- | --- | --- | --- | --- | --- |
|  |  |  |  |  |  |  |  |  |  |  | 1 | | 2 | | | | | 3 | | | | | | | 4 | | | 5 | | | 6 | | | | | 1 | | | | 2 | | | 3 |
| Chepulis 2020 | Y | Y | Y | Y | Y | Y | Y | Y | Y | Y | N | Y | Y | Y | X | Y | Y | Y | N | N | X | Y | Y | Y | Y | N | Y | X | X | X | Y | Y | Y | X | Y | Y | N | Y | Y | Y | Y | Y | Y |
| Marshall 2020 | Y | Y | Y | Y | Y | N | Y | Y | N | N | Y | Y | Y | Y | X | N | Y | Y | Y | N | X | Y | Y | Y | Y | N | Y | X | Y | Y | X | Y | Y | X | Y | Y | Y | Y | Y | Y | Y | Y | Y |
| Walker 2020 | Y | Y | Y | Y | Y | Y | Y | Y | Y | N | Y | Y | Y | Y | X | N | Y | Y | N | N | X | Y | Y | Y | Y | N | N | Y | X | X | X | Y | Y | X | Y | Y | Y | Y | Y | Y | Y | Y | Y |
| Horsburgh 2019 | Y | Y | Y | Y | Y | Y | Y | Y | Y | N | N | Y | Y | Y | X | Y | Y | Y | Y | Y | X | Y | Y | Y | Y | N | Y | X | X | X | Y | Y | Y | X | Y | Y | N | Y | Y | Y | Y | Y | Y |
| Kharjul 2019 | Y | Y | Y | Y | Y | Y | Y | Y | Y | N | N | Y | Y | Y | X | N | Y | Y | N | N | X | Y | Y | Y | Y | Y | Y | X | N | Y | Y | Y | N | X | N | Y | N | Y | Y | Y | Y | Y | Y |
| Xie 2019 | Y | Y | Y | Y | Y | Y | Y | Y | N | N | N | Y | Y | Y | X | N | Y | Y | Y | N | X | Y | Y | Y | Y | N | Y | Y | Y | Y | N | Y | Y | X | Y | Y | Y | Y | Y | Y | Y | Y | Y |
| Gatwood 2018 | Y | Y | Y | Y | Y | Y | Y | Y | Y | N | Y | Y | Y | Y | X | N | Y | Y | Y | Y | X | Y | Y | Y | Y | N | Y | X | Y | Y | Y | Y | Y | X | Y | Y | Y | Y | Y | Y | Y | Y | Y |
| McGovern 2018 | Y | Y | Y | Y | Y | Y | Y | Y | Y | N | Y | Y | Y | Y | X | N | Y | Y | Y | N | X | Y | Y | Y | Y | N | Y | X | Y | Y | X | Y | Y | X | Y | Y | Y | Y | Y | Y | Y | Y | Y |
| Taira 2017 | Y | Y | Y | Y | Y | Y | Y | Y | Y | N | Y | Y | Y | Y | X | N | Y | N | N | N | X | Y | Y | Y | Y | N | Y | X | Y | Y | Y | Y | Y | X | Y | Y | Y | Y | Y | Y | Y | Y | Y |
| Lee 2017 | Y | Y | Y | Y | Y | Y | Y | Y | Y | N | Y | Y | Y | Y | Y | X | X | Y | N | N | X | Y | Y | Y | Y | N | N | Y | X | X | Y | Y | Y | Y | Y | Y | Y | Y | Y | Y | Y | Y | Y |
| Ying 2017 | Y | Y | Y | Y | Y | N | Y | Y | Y | N | Y | Y | Y | Y | Y | N | Y | Y | N | N | X | Y | Y | Y | Y | N | Y | X | X | X | Y | Y | Y | X | Y | Y | Y | Y | Y | Y | Y | Y | Y |
| Sutton 2017 | Y | Y | Y | Y | Y | Y | Y | Y | N | N | N | Y | Y | Y | X | N | Y | Y | Y | N | X | Y | Y | Y | Y | N | Y | X | Y | Y | X | Y | Y | X | N | Y | Y | Y | Y | Y | Y | Y | Y |
| Lin 2017 | Y | Y | Y | Y | Y | Y | Y | Y | Y | N | Y | Y | Y | N | X | N | Y | Y | Y | N | X | Y | Y | Y | Y | N | Y | X | N | Y | Y | Y | Y | X | Y | Y | Y | Y | Y | Y | Y | Y | Y |
| Fernández 2017 | Y | Y | Y | Y | Y | N | Y | Y | Y | N | Y | Y | Y | N | X | N | Y | Y | N | N | X | Y | Y | Y | Y | N | N | Y | N | N | Y | Y | Y | X | Y | Y | Y | Y | Y | Y | Y | Y | Y |
| Reynolds 2016 | Y | Y | Y | Y | Y | Y | Y | Y | Y | Y | Y | Y | Y | Y | X | N | Y | Y | Y | N | X | Y | Y | Y | Y | N | Y | X | Y | Y | Y | Y | Y | X | Y | Y | Y | Y | Y | Y | Y | Y | Y |
| Patel 2016 | Y | Y | Y | Y | Y | Y | Y | Y | Y | N | Y | Y | Y | Y | X | N | Y | Y | N | Y | X | Y | Y | Y | Y | N | Y | Y | N | N | Y | Y | Y | X | Y | Y | Y | Y | Y | Y | Y | Y | Y |
| Lo-Ciganic 2016 | Y | Y | Y | Y | Y | Y | Y | Y | N | Y | Y | Y | Y | Y | X | N | Y | Y | Y | N | X | Y | Y | Y | Y | N | Y | X | Y | Y | Y | Y | Y | X | N | Y | Y | Y | Y | Y | Y | Y | Y |
| Calip 2015 | Y | Y | Y | Y | Y | Y | Y | Y | Y | N | Y | Y | Y | Y | X | N | Y | Y | N | N | X | Y | Y | Y | Y | N | Y | X | N | Y | Y | Y | Y | X | Y | Y | Y | Y | Y | Y | Y | Y | Y |
| Chong 2014 | Y | Y | Y | Y | Y | Y | Y | Y | Y | N | Y | Y | Y | Y | X | Y | Y | Y | N | N | X | Y | Y | Y | Y | Y | Y | X | N | Y | Y | Y | Y | X | Y | Y | Y | Y | Y | Y | Y | Y | Y |
| Juarez 2014 | Y | Y | Y | Y | Y | N | Y | Y | N | Y | Y | Y | Y | Y | X | N | Y | N | N | N | X | Y | Y | Y | Y | N | Y | X | N | Y | X | Y | Y | X | Y | Y | Y | Y | Y | Y | Y | Y | Y |
| Langley 2014 | Y | Y | Y | Y | Y | Y | Y | Y | Y | N | N | Y | Y | Y | X | N | Y | N | N | N | X | Y | Y | Y | Y | N | Y | X | N | Y | Y | Y | Y | X | Y | Y | Y | Y | Y | Y | Y | Y | Y |
| Rolnick 2013 | Y | Y | Y | Y | Y | Y | Y | Y | Y | Y | Y | Y | Y | Y | X | N | Y | Y | Y | N | X | Y | Y | Y | Y | N | Y | X | N | Y | Y | Y | Y | X | Y | Y | Y | Y | Y | Y | Y | Y | Y |
| Raebel 2012 | Y | Y | Y | Y | Y | Y | Y | Y | Y | N | Y | Y | Y | Y | X | N | Y | Y | Y | N | X | Y | Y | Y | Y | N | Y | Y | N | Y | Y | Y | Y | X | Y | Y | Y | Y | Y | Y | Y | Y | Y |
| Adeyemi 2012 | Y | Y | Y | Y | Y | Y | Y | Y | Y | N | Y | Y | Y | Y | X | N | Y | Y | N | N | X | Y | Y | Y | Y | N | Y | X | N | Y | Y | Y | Y | X | Y | Y | Y | Y | Y | Y | Y | Y | Y |
| Egede 2011 | Y | Y | Y | Y | Y | Y | Y | Y | Y | Y | Y | Y | Y | Y | Y | N | Y | Y | N | N | X | Y | Y | Y | Y | N | Y | X | Y | Y | Y | Y | Y | Y | Y | Y | Y | Y | Y | Y | Y | Y | Y |
| Zhu 2011 | Y | Y | Y | Y | Y | N | Y | Y | Y | Y | Y | Y | Y | Y | X | N | Y | Y | Y | N | X | Y | Y | Y | Y | N | Y | X | N | Y | Y | Y | Y | X | Y | Y | Y | Y | Y | Y | Y | Y | Y |
| Gebregziabher 2011 | Y | Y | Y | Y | Y | Y | Y | Y | Y | N | Y | Y | Y | Y | X | Y | Y | Y | Y | N | X | Y | Y | Y | Y | N | Y | X | N | Y | Y | Y | Y | X | Y | Y | Y | Y | Y | Y | Y | Y | Y |
| Osborn 2011 | Y | Y | Y | Y | Y | Y | Y | Y | N | N | N | Y | N | N | X | N | Y | N | N | N | X | Y | Y | Y | Y | N | N | Y | X | X | X | Y | Y | X | Y | Y | Y | Y | Y | Y | Y | Y | Y |
| Yang 2009 | Y | Y | Y | Y | Y | Y | Y | Y | Y | N | Y | Y | Y | Y | X | Y | Y | Y | Y | Y | X | Y | Y | Y | Y | N | Y | X | Y | Y | Y | Y | Y | X | Y | Y | Y | Y | Y | Y | Y | Y | Y |
| Trinacty 2009 | Y | Y | Y | Y | Y | Y | Y | Y | Y | N | Y | Y | Y | Y | X | N | Y | Y | Y | N | X | Y | Y | Y | N | N | N | Y | N | Y | Y | Y | Y | X | Y | Y | Y | Y | Y | Y | Y | Y | Y |
| Adams 2008 | Y | Y | Y | Y | Y | Y | Y | Y | Y | N | Y | Y | Y | Y | X | N | Y | Y | Y | N | X | Y | Y | Y | Y | N | N | Y | Y | Y | X | Y | Y | X | Y | Y | Y | Y | Y | Y | Y | Y | Y |
| Shenolikar 2006 | Y | Y | Y | Y | Y | Y | Y | Y | N | Y | Y | Y | Y | Y | X | N | Y | Y | Y | Y | X | Y | Y | Y | Y | N | Y | X | N | Y | X | Y | Y | X | Y | Y | Y | Y | Y | Y | Y | Y | Y |
| Lee 2005 | Y | Y | Y | Y | Y | Y | Y | Y | Y | N | N | Y | Y | Y | X | N | Y | Y | N | N | X | Y | Y | Y | Y | N | Y | Y | N | Y | Y | Y | Y | X | Y | Y | Y | Y | Y | Y | Y | Y | Y |
| Key | Y = yes, N = no, X = not applicable | | | | | | | | | | | | | | | |  |  |  |  |  |  |  |  |  |  |  |  |  |  |  |  |  |  |  |  |  |  |  |  |  |  |  |
