# Supplementary material for: Ethnic disparities in medication adherence? A systematic review examining the association between ethnicity and antidiabetic medication adherence
Source: PLoS One. 2023 Feb 22;18(2):e0271650. doi: 10.1371/journal.pone.0271650 (PMC9946219; doi:10.1371/journal.pone.0271650)
Supplement: S4 Table — (DOCX) [file pone.0271650.s004.docx]

**Table S4. Results of the risk of bias assessment of cross-sectional studies**

| **Author, year** | **Critical bias score** | | | | | | | |
| --- | --- | --- | --- | --- | --- | --- | --- | --- |
|  | **1** | **2** | **3** | **4** | **5** | **6** | **7** | **8** |
| Abdullah 2019 | Y | Y | X | Y | Y | Y | Y | Y |
| Jannoo 2019 | Y | Y | X | Y | N | N | Y | N |
| Jaam 2018 | Y | Y | X | Y | Y | Y | Y | Y |
| Gomes 2016 | Y | Y | X | Y | Y | Y | N | Y |
| Al-Haj 2016 | Y | Y | X | Y | Y | Y | Y | Y |
| Lopez 2014 | Y | Y | X | Y | N | N | Y | N |
| Trief 2022 | Y | Y | X | Y | Y | Y | Y | Y |
| Nasruddin 2021 | Y | Y | X | Y | Y | Y | Y | Y |
| Key | Y = yes, N = no, X = not applicable | | | | | | | |
