# Supplementary material for: Ethnic disparities in medication adherence? A systematic review examining the association between ethnicity and antidiabetic medication adherence
Source: PLoS One. 2023 Feb 22;18(2):e0271650. doi: 10.1371/journal.pone.0271650 (PMC9946219; doi:10.1371/journal.pone.0271650)
Supplement: S5 Table — (DOCX) [file pone.0271650.s005.docx]

**S5 Table. Data extraction form**

| Citation, Setting, Study design | Aim | Population | Interventions | | Outcomes measured | Medication adherence measures | Key findings | Limitations of the study |
| --- | --- | --- | --- | --- | --- | --- | --- | --- |
|  |  |  | Antidiabetic medications | Other medications |  |  |  |  |
|  |  | Characteristics of included patients:  Sample size:  Mean/median age± SD/IQR:  Gender/sex:  Ethnicity data, how is reported: |  |  |  |  |  |  |
