# Supplementary material for: Ethnic disparities in medication adherence? A systematic review examining the association between ethnicity and antidiabetic medication adherence
Source: PLoS One. 2023 Feb 22;18(2):e0271650. doi: 10.1371/journal.pone.0271650 (PMC9946219; doi:10.1371/journal.pone.0271650)
Supplement: S1 Text — (DOCX) [file pone.0271650.s006.docx]

**S1 Text: Database search terms**

MEDLINE, Embase and CINAHL Search Strategy

medication adherence.mp. OR

medication compliance.mp. OR

medication concordance.mp. OR

medication persistence.mp. OR

adherence.mp. OR

treatment adherence.mp. OR

And

diabetes mellitus.mp. OR

diabetes Mellitus, Type 1/ OR

diabetes Mellitus, Type 2/ OR

diabetes.mp. OR

insulin dependent diabetes mellitus.mp. OR

non-insulin dependent diabetes mellitus.mp.

And

glycaemic control.mp. OR

glycated Hemoglobin A.mp. OR

Hemoglobin A, Glycated.mp. OR

Hb A1c.mp. OR

Glycosylated Hemoglobin A.mp.

OR

Ethnic Groups/ OR

ethnicity.mp. OR

race.mp. or Continental Population Groups/ OR

Black.mp. or African Continental Ancestry Group/ OR

White.mp. or European Continental Ancestry Group/ OR

Caucasian/ OR

African American.mp. OR

Hispanic.mp. OR

Asian.mp. or Asian Continental Ancestry Group/ OR

Hispanic Americans/ OR

Arabs.mp. OR

Mexican American.mp. OR

Latino.mp. OR

Population Groups/ OR

Minority Groups/

PsycINFO Search Strategy

exp Treatment Compliance/ OR

medication adherence.mp. OR

medication compliance.mp. OR

medication persistence.mp. OR

medication concordance.mp.

AND

diabetes mellitus/ OR

exp diabetes/ OR

type 2 diabetes.mp. OR

type 1 diabetes.mp. OR

insulin dependent diabetes mellitus.mp. OR

non-insulin dependent diabetes mellitus.mp.

AND

glycaemic control.mp. OR

Glycated Hemoglobin A.mp. OR

Hemoglobin A, Glycated.mp. OR

Hb A1c.mp. OR

Glycosylated Hemoglobin A.mp.

OR

exp Ethnic Identity/ OR

ethnicity.mp. OR

race.mp. OR

exp blacks/ OR

African American.mp. OR

exp whites/ OR

Caucasians.mp. OR

exp Asians/ OR

exp "Latinos/Latinas"/ OR

exp Arabs/ OR

exp European Cultural Groups/
